# Supplementary material for: Multimodal joint deconvolution and integrative signature selection in proteomics
Source: Commun Biol. 2024 Apr 24;7:493. doi: 10.1038/s42003-024-06155-z (PMC11043077; doi:10.1038/s42003-024-06155-z)
Supplement: Supplementary file 3 — Description of Additional Supplementary Files [file 42003_2024_6155_MOESM3_ESM.pdf]

Description of Additional Supplementary Files

File name: Supplementary Data 1
Description: The source data for Figure 2.

File name: Supplementary Data 2
Description: The source data for Figure 3.

File name: Supplementary Data 3
Description: The source data for Figure 3.

File name: Supplementary Data 4
Description: The source data for Figure 4.

File name: Supplementary Data 5
Description: The source data for Figure 5.
